# Structural Features Defining NF-κB Inhibition by Lignan-Inspired Benzofurans and Benzothiophenes

**DOI:** 10.3390/biom10081131

**Published:** 2020-07-31

**Authors:** Toan Dao-Huy, Simone Latkolik, Julia Bräuer, Andreas Pfeil, Hermann Stuppner, Michael Schnürch, Verena M. Dirsch, Marko D. Mihovilovic

**Affiliations:** 1 Institute of Applied Synthetic Chemistry, TU Wien, Getreidemarkt 9/163, 1060 Vienna, Austria; toan.daohuy@hust.edu.vn (T.D.-H.); marko.mihovilovic@tuwien.ac.at (M.D.M.); 2 Department of Pharmaceutical and Pesticide Technology, School of Chemical Engineering, Hanoi University of Science and Technology, Dai Co Viet 1, Hai Ba Trung dist., Hanoi 10000, Vietnam; 3 Department of Pharmacognosy, University of Vienna, Althanstraße 14, 1090 Vienna, Austria; slatkolik@gmail.com (S.L.); ju.braeuer@gmail.com (J.B.); and.pfeil@gmail.com (A.P.); 4 Institute of Pharmacy/Pharmacognosy, Center for Molecular Biosciences Innsbruck, University of Innsbruck, Innrain 80/82, 6020 Innsbruck, Austria; hermann.stuppner@uibk.ac.at

**Keywords:** NF-κB inhibition, natural product, lignans, cross-coupling, C-H activation, direct arylation

## Abstract

A series of 2-arylbenzofurans and 2-arylbenzothiophenes was synthesized carrying three different side chains in position five. The synthesized compounds were tested for NF-κB inhibition to establish a structure activity relationship. It was found that both, the side chain in position five and the substitution pattern of the aryl moiety in position two have a significant influence on the inhibitory activity.

## 1. Introduction

Inflammation is a protective host response to infection or tissue damage (including stress or dyshomeostasis) [1,2]. Whereas an acute response resulting in the elimination of the noxious agent is beneficial, long lasting chronic inflammatory states contribute to the development of many pathologies like autoimmune, metabolic, cardiovascular and neurodegenerative diseases or cancer [2,3], PAMPs (pathogen-associated molecular patterns) as well as DAMPs (damage-associated molecular patterns) activate pattern recognition receptors (PRR) that transduce signals to NF-κB signaling pathways [2], which play a pivotal role in chronic and acute inflammation [4]. Thus, dampen NF-κB signaling will interfere with an inflammatory response.

**Figure 1:**
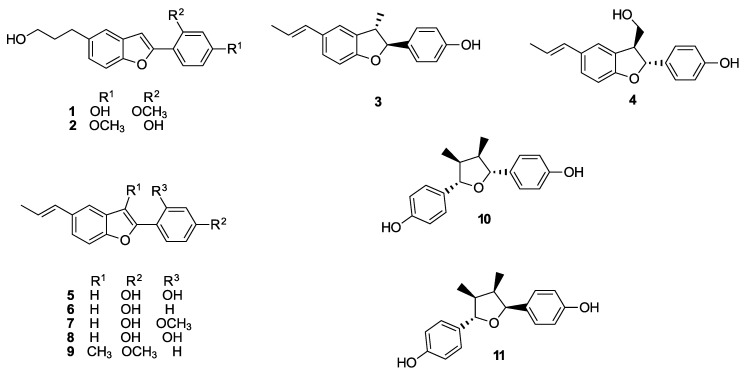
Lignan derivatives found in DCM extract of Rhatani root.

*Krameria lappacea* (Dombey), Krameriaceae, is a tropical perennial shrub growing across South America. The extract of the Rhatany root was introduced into European medicine over 200 years ago as a remedy against stomach aches, diarrhea, menstrual problems, nose bleeds and oropharyngeal inflammation [5,6]. In a study on constituents of the Rhatany root, the group of Stuppner isolated eleven lignans from the dichloromethane extract of the root (Figure 1) [7].

These isolated lignan derivatives were pharmacologically characterized in topical anti-inflammatory in vivo experiments [8]. Two of the most potent compounds, 2-(2-hydroxy-4-methoxyphenyl)-5-(3-hydroxypropyl)benzofuran **1** and (+)-conocarpan **3** (Figure 1) inhibited edema development and infiltration by neutrophils time-dependently and comparably to indomethacin. In addition, all lignans were tested in vitro for their potential to inhibit the activation of the NF-κB signaling pathway and the activity of the pro-inflammatory enzymes COX-1, COX-2, 5-LO and mPGES-1. Determination of the IC_50_ values for all compounds showed that inhibition of NF-κB is the most relevant mechanism likely contributing to the observed in vivo activity [8].

## 2. Materials and Methods

### 2.1. Chemical Synthesis

#### 2.1.1. Synthesis of 5-Allylbenzo[*b*]furan (**14**)

From 5-bromobenzo[*b*]furan **12**: Compound **12** (98 mg, 0.5 mmol), allyl-B(pin) (140 µL, 0.75 mmol, 1.5 equiv.), K_2_CO_3_ (138 mg, 1.0 mmol, 2.0 equiv.) and Pd(PPh_3_)_2_Cl_2_ (18 mg, 0.025 mmol, 5 mol %) was mixed in 1 mL DMAc at 100 °C and stirred for 24 h. The reaction mixture was then cooled to room temperature and filtered through celite, washed with EtOAc. The product was washed with saturated aq. NH_4_Cl and a last time with 10 mL brine then dried over Na_2_SO_4_ and the solvent was evaporated under reduced pressure to obtain coupling product **14** (67 mg, colorless oil), 85% yield.

From 5-chlorobenzo[*b*]furan **17**: Compound **17** (76 mg, 0.5 mmol), allyl-B(pin) (126 mg, 140 µL, 0.75 mmol, 1.5 equiv.), Cs_2_CO_3_ (326 mg, 1.0 mmol, 2.0 equiv.), SPhos (21 mg, 0.05 mmol, 10 mol %) and Pd_2_(dba)_3_ (23 mg, 0.025 mmol, 5 mol %) was mixed in 2 mL DMAc and at 100 °C and stirred for 6 h. The reaction mixture was then cooled to room temperature and filtered through celite and washed with EtOAc. The product was washed with saturated aq. NH_4_Cl and a last time with 10 mL brine then dried over Na_2_SO_4_ and the solvent was evaporated under reduced pressure to obtain coupling product **14** (54 mg, colorless oil), 68% yield.

^1^H-NMR (200 MHz, CDCl_3_) δ (ppm) 3.51 (d, *J* = 6.7 Hz, 2H), 5.08–5.17 (m, 2H), 5.95–6.15 (m, 1H), 6.73–6.75 (m, 1H), 7.16 (d, *J* = 8.6 Hz, 1H), 7.44–7.48 (m, 2H), 7.62 (d, *J* = 2.1 Hz, 1H). ^13^C-NMR (50 MHz, CDCl_3_) δ (ppm) 40.1, 106.4, 111.1, 115.6, 120.7, 125.1, 127.6, 134.5, 138.0, 145.1, 153.7. MS analyst, *m*/*z* (Int.) 158(100), 157(46), 129(82), 128(50), 115(18), 102(10), 89(10), 77(22), 63(15).

#### 2.1.2. General Procedure of C-H Activation Reaction on 5-Chlorobenzo[*b*]furan (**17**)

Procedure A: Benzo-fused starting material (1.0 mmol, 1.0 equiv.), aryl bromide (1.5 mmol, 1.5 equiv.), cesium pivalate (350 mg, 1.5 mmol, 1.5 equiv.), Pd(OAc)_2_ (9 mg, 0.04 mmol, 0.04 equiv.) and SPhos (16.4 mg, 0.08 mmol, 0.08 equiv.) was mixed in 2 mL of degassed DMAc. The mixture was stirred at 140 °C for 24 h in argon atmosphere. The reaction mixture was cooled to room temperature and then diluted with 15 mL diethyl ether or ethyl acetate (depending on the polarity of the product) and filtered through a pad of celite. The organic phase was washed with saturated NH_4_Cl solution, once with brine and then dried over Na_2_SO_4_. The solvent was removed under reduced pressure. Purification was performed on silica gel eluting with LP or LP/EtOAc mixtures (depending on the polarity of the product).

#### 2.1.3. 5-Chloro-2-(4-(methoxymethoxy)phenyl)benzo[*b*]furan (**18a**)

Prepared according to the general procedure A. mp 138–141 °C. ^1^H-NMR (400 MHz, CDCl_3_) δ (ppm) = 3.50 (s, 3H), 5.22 (s, 2H), 6.82 (s, 1H), 7.09–7.13 (m, 2H), 7.19 (dd, *J* = 8.7, 2.1 Hz, 1H), 7.39 (d, *J* = 8.7 Hz, 1H), 7.50 (d, *J* = 2.1 Hz, 1H), 7.76 (m, 2H). ^13^C-NMR (100 MHz, CDCl_3_) δ (ppm) = 56.1, 94.3, 99.5, 111.9, 116.5 (2C), 120.1, 123.8, 123.9, 126.5 (2C), 128.4, 130.8, 153.1, 157.4, 157.9. HR-MS analyst [M + H]^+^ *m*/*z* (predicted) = 289.0631, *m*/*z* (measured) = 289.0636, difference = −1.90 ppm.

#### 2.1.4. 5-Chloro-2-(4-methoxyphenyl)benzo[*b*]furan (**18c**)

Prepared according to the general procedure A. mp 144–146 °C. ^1^H-NMR (200 MHz, CDCl_3_) δ (ppm) = 3.86 (s, 3H), 6.81 (s, 1H), 6.95–7.00 (m, 2H), 7.18 (dd, *J* = 8.7, 2.1 Hz, 1H), 7.39 (d, *J* = 8.4 Hz, 1H), 7.50 (d, *J* = 2.1 Hz, 1H), 7.76–7.80 (m, 2H). ^13^C-NMR (50 MHz, CDCl_3_) δ (ppm) = 55.4, 99.2, 111.9, 114.3 (2C), 120.1, 122.8, 123.8, 126.6 (2C), 128.3, 130.9, 153.1, 157.5, 160.3.

#### 2.1.5. 5-Chloro-2-(3,5-dimethoxyphenyl)benzo[*b*]furan (**18d**)

Prepared according to the general procedure A. ^1^H-NMR (400 MHz, CDCl_3_) δ (ppm) = 3.86 (s, 6H), 6.48 (t, *J* = 2.3 Hz, 1H), 6.93 (d, *J* = 0.8 Hz, 1H), 6.99 (d, *J* = 2.3 Hz, 2H), 7.23 (dd, *J* = 8.7, 2.1 Hz, 1H), 7.42 (d, *J* = 8.7 Hz, 1H), 7.53 (d, *J* = 2.1 Hz, 1H). ^13^C-NMR (100 MHz, CDCl_3_) δ (ppm) = 55.5 (2C), 101.3, 101.4 (2C), 103.2, 112.1, 120.5, 124.5, 128.5, 130.5, 131.7, 153.2, 157.2, 161.2 (2C).

#### 2.1.6. 5-Chloro-2-phenylbenzo[*b*]furan (**18e**)

Prepared according to the general procedure A. mp 125–128 °C. ^1^H-NMR (200 MHz, CDCl_3_) δ (ppm) = 6.96 (s, 1H), 7.20–7.55 (m, 6H), 7.81–7.87 (m, 2H). ^13^C-NMR (50 MHz, CDCl_3_) δ (ppm) = 100.8, 112.1, 120.4, 124.4, 125.0 (2C), 128.5, 128.8 (2C), 129.0, 130.0, 130.6, 153.2, 157.4.

#### 2.1.7. 5-Chloro-2-(4-fluorophenyl)benzo[*b*]furan (**18f**)

Prepared according to the general procedure A. mp 119–122 °C. ^1^H-NMR (200 MHz, CDCl_3_) δ (ppm) = 6.86 (s, 1H), 7.09–7.24 (m, 3H), 7.38–7.52 (m, 2H), 7.76–7.83 (m, 2H). ^13^C-NMR (50 MHz, CDCl_3_) δ (ppm) = 100.5, 112.1, 116.0 (d, ^2^*J*_C-F_ = 21.9 Hz), 120.4, 124.4, 126.3 (d, ^4^*J*_C-F_ = 3.4 Hz), 126.9 (d, ^3^*J*_C-F_ = 8.3 Hz, 2C), 128.6, 130.5, 153.2, 156.5, 163.1 (d, ^1^*J*_C-F_ = 247.9 Hz).

#### 2.1.8. 5-Chloro-2-(4-(difluoromethyl)phenyl)benzo[*b*]furan (**18g**)

Prepared according to the general procedure A. mp 121–124 °C. ^1^H-NMR (200 MHz, CDCl_3_) δ (ppm) = 6.69 (t, *J_H-F_* = 56.4 Hz, 1H), 7.02 (s, 1H), 7.26 (dd, *J* = 8.7, 2.1 Hz, 1H), 7.44 (d, *J* = 8.7 Hz, 1H), 7.55–7.62 (m, 3H), 7.90–7.93 (m, 2H). ^13^C-NMR (50 MHz, CDCl_3_) δ (ppm) = 102.1, 112.2, 114.4 (t, ^1^*J*_C-F_ = 237.6 Hz), 120.7, 125.0, 125.2 (2C), 126.1 (t, ^3^*J*_C-F_ = 6.1 Hz), 128.7, 130.3, 132.2, 134.6 (t, ^2^*J*_C-F_ = 22.5 Hz), 153.4, 156.1. HR-MS analyst [M + H]^+^ *m*/*z* (predicted) = 279.0388, *m*/*z* (measured) = 279.0392, difference = −1.62 ppm.

#### 2.1.9. 5-Chloro-2-(2-chlorophenyl)benzo[*b*]furan (**18h**)

Prepared according to the general procedure A. ^1^H-NMR (200 MHz, CDCl_3_) δ (ppm) = 7.26–7.54 (m, 6H), 7.61 (d, *J* = 1.9 Hz, 1H), 8.04 (dd, *J* = 7.7, 1.8 Hz, 1H). ^13^C-NMR (50 MHz, CDCl_3_) δ (ppm) = 106.8, 112.0, 120.9, 125.1, 127.0, 128.4, 128.5, 129.0, 129.5, 130.4, 130.9, 131.5, 152.5, 153.4.

#### 2.1.10. 5-Chloro-2-(4-(methoxymethoxy)phenyl)benzo[*b*]thiophene (**22a**)

Prepared according to the general procedure A. mp 175–177 °C. ^1^H-NMR (400 MHz, CDCl_3_) δ (ppm) = 3.51 (s, 3H), 5.22 (s, 2H), 7.09–7.12 (m, 2H), 7.25 (dd, *J* = 8.7, 2.0 Hz, 1H), 7.34 (s, 1H), 7.60–7.63 (m, 2H), 7.69–7.71 (m, 2H). ^13^C-NMR (100 MHz, CDCl_3_) δ (ppm) = 56.1, 94.4, 116.7 (2C), 117.7, 122.8, 123.2, 124.4, 127.7, 127.8 (2C), 130.7, 137.3, 142.0, 146.1, 157.7. HR-MS analyst [M + H]^+^ *m*/*z* (predicted) = 305.0403, *m*/*z* (measured) = 305.0396, difference = 2.02 ppm.

#### 2.1.11. 5-Chloro-2-(4-methoxyphenyl)benzo[*b*]thiophene (**22c**)

Prepared according to the general procedure A. mp 163–165 °C. ^1^H-NMR (200 MHz, CDCl_3_) δ (ppm) = 3.86 (s, 3H), 6.96 (d, *J* = 8.8 Hz, 2H), 7.21–7.26 (m, 1H), 7.34 (s, 1H), 7.63 (d, *J* = 8.7 Hz, 2H), 7.68–7.72 (m, 2H). ^13^C-NMR (100 MHz, benzene-d6) δ (ppm) = 54.6, 114.5 (2C), 118.6, 122.3, 123.4, 124.5, 127.7, 127.9 (2C), 130.6, 137.4, 141.3, 146.4, 160.1. HR-MS analyst [M + H]^+^ *m*/*z* (predicted) = 275.0297, *m*/*z* (measured) = 275.0300, difference = −1.04 ppm.

#### 2.1.12. General Procedure of the Suzuki-Miyaura Coupling of Chloro Benzo-Fused Derivatives

Procedure B: 5-chloro benzo-fused (1.0 mmol, 1.0 equiv.), allylboronic acid pinacol ester (1.5 mmol, 1.5 equiv.), cesium carbonate (486 mg, 1.5 mmol, 1.5 equiv.), Pd_2_(dba)_3_ (45 mg, 0.05 mmol, 0.05 equiv.) and SPhos (41 mg, 0.1 mmol, 0.1 equiv.) was mixed in 2 mL of dried dioxane. The mixture was stirred at 100 °C for 5 h in argon atmosphere. The reaction mixture was cooled to room temperature and then diluted with 15 mL diethyl ether or ethyl acetate (depending on the polarity of the product) and filtered through a pad of celite. The organic phase was washed with saturated NH_4_Cl solution, once with brine and then dried over Na_2_SO_4_. The solvent was removed under reduced pressure. Purification was performed on silica gel eluting with LP or LP/EtOAc mixtures (depending on the polarity of the product).

#### 2.1.13. 5-Allyl-2-(4-(methoxymethoxy)phenyl)benzo[*b*]furan (**16a**)

Prepared according to the general procedure B. mp 126–129 °C. ^1^H-NMR (400 MHz, CDCl_3_) δ (ppm) = 3.47–3.51 (m, 5H), 5.07–5.23 (m, 4H), 6.04 (ddt, *J* = 16.8, 10.1, 6.7 Hz, 1H), 6.85 (s, 1H), 7.07–7.14 (m, 3H), 7.36–7.44 (m, 2H), 7.76–7.81 (m, 2H). ^13^C-NMR (100 MHz, CDCl_3_) δ (ppm) = 40.2, 56.1, 94.4, 99.9, 110.8, 115.5, 116.5 (2C), 120.2, 124.5, 124.7, 126.3 (2C), 129.6, 134.6, 138.1, 153.6, 156.1, 157.5. HR-MS analyst [M + H]^+^ *m*/*z* (predicted) = 295.1329, *m*/*z* (measured) = 295.1323, difference = 2.1 ppm.

#### 2.1.14. 5-Allyl-2-(4-methoxyphenyl)benzo[*b*]furan (**16c**)

Prepared according to the general procedure B. mp 130–132 °C. ^1^H-NMR (400 MHz, CDCl_3_) δ (ppm) = 3.49 (d, *J* = 6.6 Hz, 2H), 3.86 (s, 3H), 5.07–5.17 (m, 2H), 6.05 (ddt, *J* = 16.8, 10.1, 6.7 Hz, 1H), 6.84 (s, 1H), 6.93–7.00 (m, 2H), 7.09 (dd, *J* = 8.4, 1.7 Hz, 1H), 7.36–7.45 (m, 2H), 7.77–7.82 (m, 2H). ^13^C-NMR (100 MHz, CDCl_3_) δ (ppm) = 40.2, 55.4, 99.6, 110.7, 114.3 (2C), 115.5, 120.2, 123.5, 124.6, 126.4 (2C), 129.7, 134.6, 138.1, 153.5, 156.3, 160.0. HR-MS analyst [M + H]^+^ *m*/*z* (predicted) = 265.1223, *m*/*z* (measured) = 265.1221, difference = 0.93 ppm.

#### 2.1.15. 5-Allyl-2-(3,5-dimethoxyphenyl)benzo[*b*]furan (**16d**)

Prepared according to the general procedure B. ^1^H-NMR (400 MHz, CDCl_3_) δ (ppm) = 3.37 (d, *J* = 6.7 Hz, 2H), 3.76 (s, 6H), 4.98–5.03 (m, 2H), 5.92 (ddt, *J* = 16.8, 10.1, 6.7 Hz, 1H), 6.37 (t, *J* = 2.3 Hz, 1H), 6.84 (d, *J* = 0.8 Hz, 1H), 6.91 (d, *J* = 2.3 Hz, 2H), 7.01 (dd, *J* = 8.4, 1.7 Hz, 1H), 7.27–7.34 (m, 2H). ^13^C-NMR (100 MHz, CDCl_3_) δ (ppm) = 40.2, 55.5, 101.2, 101.8, 103.0 (2C), 110.9, 115.6, 120.5, 125.3, 129.3, 132.3, 134.8, 138.0, 153.7, 155.0, 161.1 (2C). HR-MS analyst [M + H]^+^ *m*/*z* (predicted) = 295.1329, *m*/*z* (measured) = 295.1328, difference = 0.17 ppm.

#### 2.1.16. 5-Allyl-2-phenylbenzo[*b*]furan (**16e**)

Prepared according to the general procedure B. mp 118–120 °C. ^1^H-NMR (200 MHz, CDCl_3_) δ (ppm) = 3.37 (d, *J* = 6.5 Hz, 2H), 4.96–5.04 (m, 2H), 5.92 (ddt, *J* = 16.8, 10.1, 6.7 Hz, 1H), 6.83 (s, 1H), 7.00 (d, *J* = 8.4 Hz, 1H), 7.21–7.35 (m, 5H), 7.72–7.75 (d, *J* = 7.2 Hz, 2H). ^13^C-NMR (50 MHz, CDCl_3_) δ (ppm) = 40.2, 101.2, 110.9, 115.6, 120.5, 124.9 (2C), 125.1, 128.5, 128.8 (2C), 129.5, 130.6, 134.7, 138.1, 153.8, 156.2. HR-MS analyst [M + H]^+^ *m*/*z* (predicted) = 235.1117, *m*/*z* (measured) = 235.1119, difference = −0.8 ppm.

#### 2.1.17. 5-Allyl-2-(4-fluorophenyl)benzo[*b*]furan (**16f**)

Prepared according to the general procedure B. mp 113–115 °C. ^1^H-NMR (400 MHz, CDCl_3_) δ (ppm) = 3.50 (d, *J* = 6.7 Hz, 2H), 5.09–5.16 (m, 2H), 6.05 (ddt, *J* = 16.8, 10.1, 6.7 Hz, 1H), 6.90 (s, 1H), 7.11–7.16 (m, 3H), 7.39–7.45 (m, 2H), 7.81–7.84 (m, 2H). ^13^C-NMR (100 MHz, CDCl_3_) δ (ppm) = 40.2, 100.9, 110.9, 115.6, 115.9 (d, ^2^*J*_C-F_ = 21.9 Hz), 120.5, 125.2, 126.7 (d, ^3^*J*_C-F_ = 8.5 Hz), 126.9 (d, ^4^*J*_C-F_ = 3.0), 129.4, 134.8, 138.0, 153.7, 155.3, 162.9 (d, ^1^*J*_C-F_ = 248.7). HR-MS analyst [M + H]^+^ *m*/*z* (predicted) = 253.1023, *m*/*z* (measured) = 253.1034, difference = −4.4 ppm.

#### 2.1.18. 5-Allyl-2-(4-(difluoromethyl)phenyl)benzo[*b*]furan (**16g**)

Prepared according to the general procedure B. mp 116–118 °C. ^1^H-NMR (400 MHz, CDCl_3_) δ (ppm) = 3.50 (d, *J* = 6.6 Hz, 2H), 5.08–5.17 (m, 2H), 6.04 (ddt, *J* = 17.0, 10.4, 6.7 Hz, 1H), 6.68 (t, *J*_H-F_ = 56.4 Hz, 1H), 7.04 (s, 1H), 7.15 (dd, *J* = 8.4, 1.6 Hz, 1H), 7.41–7.60 (m, 4H), 7.90–7.94 (m, 2H). ^13^C-NMR (100 MHz, CDCl_3_) δ (ppm) = 40.1, 102.6, 111.0, 114.5 (t, ^1^*J*_C-F_ = 238.8 Hz), 115.7, 120.7, 125.0 (2C), 125.8, 126.1 (t, ^3^*J*_C-F_ = 6.1 Hz), 129.2, 132.8, 134.1 (t, ^2^*J*_C-F_ = 22.4 Hz), 135.0, 137.9, 153.9, 154.9. HR-MS analyst [M + H]^+^ *m*/*z* (predicted) = 285.1091, *m*/*z* (measured) = 285.1088, difference = 1.08 ppm.

#### 2.1.19. 5-Allyl-2-(2-chlorophenyl)benzo[*b*]furan (**16h**)

Prepared according to the general procedure B. ^1^H-NMR (200 MHz, CDCl_3_) δ (ppm) = 3.57 (d, *J* = 6.2 Hz, 2H), 4.90–5.03 (m, 2H), 5.95 (ddt, *J* = 17.0, 10.4, 6.7 Hz, 1H), 6.74 (d, *J* = 0.7 Hz, 1H), 7.17–7.67 (m, 6H), 7.76 (d, *J* = 8.1. Hz, 1H). ^13^C-NMR (50 MHz, CDCl_3_) δ (ppm) = 38.3, 104.5, 112.1, 116.3, 120.5, 124.4, 126.6, 129.1, 129.2, 129.5 (2C), 130.4, 130.7, 136.8, 138.0, 153.0, 157.1. HR-MS analyst [M + H]^+^ *m*/*z* (predicted) = 269.0733, *m*/*z* (measured) = 269.0730, difference = 1.11 ppm.

#### 2.1.20. 5-Allyl-2-(4-(methoxymethoxy)phenyl)benzo[*b*]thiophene (**23a**)

Prepared according to the general procedure B. mp 164–166 °C. ^1^H-NMR (400 MHz, CDCl_3_) δ (ppm) = 3.46–3.48 (m, 5H), 5.07–5.13 (m, 2H), 5.19 (s, 2H), 6.01 (ddt, *J* = 16.8, 10.1, 6.7 Hz, 1H), 7.06–7.13 (m, 3H), 7.35 (s, 1H), 7.53 (d, *J* = 0.7 Hz, 1H), 7.59–7.61 (m, 2H), 7.70 (d, *J* = 8.2 Hz, 1H). ^13^C-NMR (100 MHz, CDCl_3_) δ (ppm) = 40.2, 56.1, 94.4, 115.8, 116.7 (2C), 118.4, 122.1, 123.0, 125.3, 127.7 (2C), 128.3, 136.5, 137.2, 137.7, 141.3, 144.3, 157.4. HR-MS analyst [M + H]^+^ *m*/*z* (predicted) = 311.1100, *m*/*z* (measured) = 311.1108, difference = −2.62 ppm.

#### 2.1.21. 5-Allyl-2-(4-methoxyphenyl)benzo[*b*]thiophene (**23c**)

Prepared according to the general procedure B. mp 146–147 °C. ^1^H-NMR (200 MHz, CDCl_3_) δ (ppm) = 3.49 (d, *J* = 6.7 Hz, 2H), 3.80 (s, 3H), 5.06–5.16 (m, 2H), 6.02 (ddt, *J* = 16.8, 10.2, 6.7 Hz, 1H), 6.90–6.98 (m, 2H), 7.13 (dd, *J* = 8.2, 1.6 Hz, 1H), 7.36 (s, 1H), 7.54 (s, 1H), 7.63 (d, *J* = 8.8 Hz, 2H), 7.71 (d, *J* = 8.2 Hz, 1H). ^13^C-NMR (50 MHz, CDCl_3_) δ (ppm) = 40.2, 55.4, 114.3 (2C), 115.8, 118.0, 122.0, 122.9, 125.1, 127.2, 127.7 (2C), 136.4, 137.1, 137.7, 141.3, 144.4, 159.8. HR-MS analyst [M + H]^+^ *m*/*z* (predicted) = 281.0995, *m*/*z* (measured) = 281.1000, difference = −1.98 ppm.

#### 2.1.22. General Procedure for Hydroboration-Oxidation on Allyl Benzo-Fused Heterocycles

Procedure C: 5-allyl benzo-fused derivatives (0.5 mmol, 1.0 equiv.) was dissolved in 0.5 mL dry THF then the solution was cooled to 0 °C. A 1M solution of BH_3_^.^THF (0.5 mL, 0.5 mmol, 1.0 equiv.) was added slowly. Afterwards, the reaction solution was warmed to room temperature and stirred for 24 h. On the other hand, a solution of 3M NaOH and H_2_O_2_ 30% was mixed in ratio of 2:3 and then cooled to 0 °C. After 24 h of reaction time, the reaction was cooled again to 0 °C and then the prepared solution of NaOH and H_2_O_2_ (1.20 mL, 1.2 mmol NaOH and 7.8 mmol H_2_O_2_) was added slowly. The reaction mixture was stirred at room temperature for 4 more hours, then diluted with 5 mL diethyl ether. The organic phase was washed with a saturated NH_4_Cl solution for 3 times, once with brine and dried over Na_2_SO_4_. The solvent was removed under reduced pressure. Purification was performed on silica gel eluting with LP/EtOAc mixtures.

#### 2.1.23. 3-(2-(4-(Methoxymethoxy)phenyl)benzo[*b*]furan-5-yl)propan-1-ol (**19a**)

Prepared according to the general procedure C. mp 155–156 °C. ^1^H-NMR (200 MHz, CDCl_3_) δ (ppm) = 1.33 (s, 1H), 1.88–2.01 (m, 2H), 2.81 (t, *J* = 7.3 Hz, 2H), 3.51 (s, 3H), 3.70 (t, J = 6.4 Hz, 2H), 5.23 (s, 2H), 6.85 (s, 1H), 7.08–7.13 (m, 3H), 7.38–7.43 (m, 2H), 7.78 (d, *J* = 8.7 Hz, 2H). ^13^C-NMR (50 MHz, CDCl_3_) δ (ppm) = 32.0, 34.8, 56.1, 62.3, 94.4, 99.8 110.7, 116.5 (2C), 120.0, 124.4, 124.5, 126.3 (2C), 129.6, 136.3, 153.4, 156.1, 157.5. HR-MS analyst [M + H]^+^ *m*/*z* (predicted) = 313.1434, *m*/*z* (measured) = 313.1431, difference = 0.93 ppm.

#### 2.1.24. 3-(2-(4-Methoxyphenyl)benzo[*b*]furan-5-yl)propan-1-ol (**19c**)

Prepared according to the general procedure C. mp 150–152 °C. ^1^H-NMR (400 MHz, CDCl_3_) δ (ppm) = 1.41 (s, 1H), 1.94 (ddt, *J* = 12.9, 8.9, 6.4 Hz, 2H), 2.80 (t, *J* = 7.2 Hz, 2H), 3.70 (t, *J* = 6.4 Hz, 2H), 3.86 (s, 3H), 6.83 (d, *J* = 0.8 Hz, 1H), 6.95–7.00 (m, 2H), 7.09 (dd, *J* = 8.4, 1.8 Hz, 1H), 7.36–7.41 (m, 2H), 7.76–7.80 (m, 2H). ^13^C-NMR (100 MHz, CDCl_3_) δ (ppm) = 32.0, 34.8, 55.4, 62.3, 99.5, 110.7, 114.2 (2C), 119.9, 123.5, 124.4, 126.4 (2C), 129.7, 136.3, 153.4, 156.3, 159.9. HR-MS analyst [M + H]^+^ *m*/*z* (predicted) = 283.1329, *m*/*z* (measured) = 283.1339, difference = −3.63 ppm.

#### 2.1.25. 3-(2-(3,5-Dimethoxyphenyl)benzo[*b*]furan-5-yl)propan-1-ol (**19d**)

Prepared according to the general procedure C. mp 66–68 °C. ^1^H-NMR (400 MHz, CDCl_3_) δ (ppm) = 1.43 (s, 1H), 1.94 (ddt, *J* = 12.9, 8.9, 6.4 Hz, 2H), 2.80 (t, *J* = 7.4 Hz, 2H), 3.70 (t, *J* = 6.4 Hz, 2H), 3.87 (s, 6H), 6.47 (t, *J* = 2.2 Hz, 1H), 6.95 (s, 1H), 7.01 (d, *J* = 2.2 Hz, 2H), 7.12 (dd, *J* = 8.4, 1.6 Hz, 1H), 7.39–7.44 (m, 2H). ^13^C-NMR (100 MHz, CDCl_3_) δ (ppm) = 32.0, 34.7, 55.5 (2C), 62.2, 101.0, 101.7, 103.0 (2C), 110.9, 120.3, 125.1, 129.5, 132.3, 136.5, 153.5, 155.9, 161.1 (2C). HR-MS analyst [M + H]^+^ *m*/*z* (predicted) = 313.1434, *m*/*z* (measured) = 313.1430, difference = 1.32 ppm.

#### 2.1.26. 3-(2-Phenylbenzo[*b*]furan-5-yl)propan-1-ol (**19e**)

Prepared according to the general procedure C. mp 128–129 °C. ^1^H-NMR (400 MHz, CDCl_3_) δ (ppm) = 1.39 (s, 1H), 1.95 (ddt, *J* = 12.9, 8.9, 6.4 Hz, 2H), 2.81 (t, *J* = 7.6 Hz, 2H), 3.71 (t, *J* = 6.4 Hz, 2H), 6.97 (d, *J* = 0.8 Hz, 1H), 7.13 (dd, *J* = 8.4, 1.8 Hz, 1H), 7.33–7.47 (m, 5H), 7.84–7.87 (m, 2H). ^13^C-NMR (100 MHz, CDCl_3_) δ (ppm) = 32.0, 34.8, 62.3, 101.1, 110.9, 120.2, 124.9 (2C), 125.0, 128.5, 128.8 (2C), 129.4, 130.6, 136.5, 153.6, 156.2. HR-MS analyst [M + H]^+^ *m*/*z* (predicted) = 253.1223, *m*/*z* (measured) = 253.1220, difference = 1.05 ppm.

#### 2.1.27. 3-(2-(4-Fluorophenyl)benzo[*b*]furan-5-yl)propan-1-ol (**19f**)

Prepared according to the general procedure C. mp 124–126 °C. ^1^H-NMR (400 MHz, CDCl_3_) δ (ppm) = 1.41 (s, 1H), 1.94 (ddt, *J* = 12.9, 8.9, 6.4 Hz, 2H), 2.81 (t, *J* = 7.6 Hz, 2H), 3.72 (t, *J* = 6.4 Hz, 2H), 6.89 (d, *J* = 0.4 Hz, 1H), 7.11–7.15 (m, 3H), 7.33 (m, 2H), 7.80–7.84 (m, 2H). ^13^C-NMR (100 MHz, CDCl_3_) δ (ppm) = 32.0, 34.8, 62.3, 100.9, 110.9, 115.9 (d, ^2^*J*_C-F_ = 22.0 Hz, 2C), 120.2, 125.0, 126.7 (d, ^3^*J*_C-F_ = 8.2 Hz, 2C), 126.9 (d, ^4^*J*_C-F_ = 3.4 Hz), 129.4, 136.6, 153.6, 155.3, 162.9 (d, ^1^*J*_C-F_ = 248.6 Hz). HR-MS analyst [M + H]^+^ *m*/*z* (predicted) = 295.1329, *m*/*z* (measured) = 295.1327, difference = 0.69 ppm.

#### 2.1.28. 3-(2-(4-(Difluoromethyl)phenyl)benzo[*b*]furan-5-yl)propan-1-ol (**19g**)

Prepared according to the general procedure C. mp 127–129 °C. ^1^H-NMR (400 MHz, CDCl_3_) δ (ppm) = 1.59 (s, 1H), 1.95 (ddt, *J* = 12.9, 8.9, 6.4 Hz, 2H), 2.82 (t, *J* = 7.6 Hz, 2H), 3.71 (t, *J* = 6.4 Hz, 2H), 6.68 (t, *J* = 56.4 Hz, 1H), 7.04 (s, 1H), 7.16 (dd, *J* = 8.4, 1.8 Hz, 1H), 7.41–7.45 (m, 2H), 7.58 (d, *J* = 8.2 Hz, 2H), 7.92 (d, *J* = 8.5 Hz, 2H). ^13^C-NMR (100 MHz, CDCl_3_) δ (ppm) = 32.0, 34.7, 62.2, 102.5, 111.0, 114.5 (t, ^1^*J*_C-F_ = 238.8 Hz), 120.5, 125.0 (2C), 125.6, 126.1 (t, ^3^*J*_C-F_ = 6.1 Hz), 129.1, 132.8, 134.1 (t, ^2^*J*_C-F_ = 22.4 Hz), 136.8, 153.8, 154.9. HR-MS analyst [M + H]^+^ *m*/*z* (predicted) = 303.1191, *m*/*z* (measured) = 303.1185, difference = 2.17 ppm.

#### 2.1.29. 3-(2-(2-Chlorophenyl)benzo[*b*]furan-5-yl)propan-1-ol (**19h**)

Prepared according to the general procedure C. ^1^H-NMR (400 MHz, CDCl_3_) δ (ppm) = 1.39 (s, 1H), 1.95 (ddt, *J* = 12.9, 8.9, 6.4 Hz, 2H), 2.81 (t, *J* = 7.6 Hz, 2H), 3.71 (t, *J* = 6.4 Hz, 2H), 6.61 (d, *J* = 1.9 Hz, 1H), 7.26–7.54 (m, 6H), 7.83 (dd, *J* = 7.7, 1.8 Hz, 1H). ^13^C-NMR (100 MHz, CDCl_3_) δ (ppm) = 31.8, 36.0, 64.2, 104.1, 113.3, 120.4, 124.0, 127.4, 127.8, 128.4, 129.1, 129.1, 131.9, 132.5, 134.3, 151.8, 153.5. HR-MS analyst [M + H]^+^ *m*/*z* (predicted) = 287.0833, *m*/*z* (measured) = 287.0826, difference = 2.71 ppm.

#### 2.1.30. 3-(2-(4-(Methoxymethoxy)phenyl)benzo[*b*]thiophen-5-yl)propan-1-ol (**24a**)

Prepared according to the general procedure C. mp 170–172 °C. ^1^H-NMR (400 MHz, CDCl_3_) δ (ppm) = 1.44 (s, 1H), 1.95 (ddt, *J* = 12.9, 8.9, 6.4 Hz, 2H), 2.82 (t, *J* = 7.4 Hz, 2H), 3.50 (s, 3H), 3.71 (t, *J* = 6.4 Hz, 2H), 5.22 (s, 2H), 7.07–7.16 (m, 3H), 7.38 (s, 1H), 7.57 (d, *J* = 0.9 Hz, 1H), 7.57–7.64 (m, 2H), 7.72 (d, *J* = 8.2 Hz, 1H). ^13^C-NMR (100 MHz, CDCl_3_) δ (ppm) = 32.0, 34.5, 56.1, 62.2, 94.4, 116.6 (2C), 118.3, 122.1, 122.8, 125.1, 127.7 (2C), 128.3, 137.0, 138.2, 141.2, 144.3, 157.4. HR-MS analyst [M + H]^+^ *m*/*z* (predicted) = 329.1206, *m*/*z* (measured) = 329.1211, difference = −1.91 ppm.

#### 2.1.31. 3-(2-(4-Methoxyphenyl)benzo[*b*]thiophen-5-yl)propan-1-ol (**24c**)

Prepared according to the general procedure C. mp 151–153 °C. ^1^H-NMR (200 MHz, CDCl_3_) δ (ppm) = 1.29 (s, 1H), 1.96 (ddt, *J* = 12.9, 8.9, 6.4 Hz, 2H, H-2″), 2.82 (t, *J* = 7.4 Hz, 2H, H-1″), 3.71 (t, *J* = 6.4 Hz, 2H), 3.85 (s, 3H), 6.94–6.96 (m, 2H), 7.08–7.16 (m, 1H), 7.36–7.38 (m, 1H), 7.57 (s, 1H), 7.62–7.64 (m, 2H), 7.71 (d, *J* = 8.2 Hz, 1H). ^13^C-NMR (50 MHz, C′DCl_3_) δ (ppm) = 32.0, 34.5, 55.4, 62.3 (C-3″), 114.4 (2C), 118.0, 122.1, 122.7, 125.0, 127.71, 127.72 (2C), 136.9, 138.2, 141.2, 144.4, 159.8. HR-MS analyst [M + H]^+^ *m*/*z* (predicted) = 299.1100, *m*/*z* (measured) = 299.1072, difference = 9.57 ppm.

#### 2.1.32. General Procedure for Isomerization on Allyl Benzo-Fused Heterocycles

Procedure D: 5-allyl benzo-fused derivatives (0.5 mmol, 1.0 equiv.), Pd(dba)_2_ (5.8 mg, 0.01 mmol, 0.02 equiv.), P(*^t^*Bu_3_)^.^HBF_4_ (5.8 mg, 0.02 mmol, 0.04 equiv.) and *^i^*PrCOCl (10 µL, 10.6 mg, 0.1 mmol, 0.2 equiv.) was mixed in 1 mL of degassed DMAc. The mixture was stirred at 100 °C for 6 h in argon atmosphere. The reaction mixture was cooled to room temperature and then diluted with 15 mL ethylacetate and filtered through a pad of celite. The organic phase was washed with a saturated NH_4_Cl solution for 3 times, once with brine and dried over Na_2_SO_4_. The solvent was removed under reduced pressure. Purification was performed on silica gel eluting with LP or LP/EtOAc mixtures (depending on the polarity of the product).

#### 2.1.33. (*E*)-2-(4-(Methoxymethoxy)phenyl)-5-(prop-1-en-1-yl)benzo[*b*]furan (**20a**)

Prepared according to the general procedure D. mp 151–153 °C. ^1^H-NMR (200 MHz, CDCl_3_) δ (ppm) = 1.90 (dd, *J* = 6.6, 1.5 Hz, 3H), 3.50 (s, 3H), 5.22 (s, 2H), 6.20 (dq, *J* = 15.6, 6.5 Hz, 1H), 6.48 (dd, *J* = 15.7, 1.3 Hz, 1H), 6.85 (s, 1H), 7.09–7.12 (m, 2H), 7.25–7.27 (m, 1H), 7.39–7.47 (m, 2H), 7.76–7.79 (m, 2H). ^13^C-NMR (50 MHz, CDCl_3_) δ (ppm) = 18.5, 56.1, 94.4, 100.1, 110.9, 116.5 (2C), 117.7, 122.1, 124.4, 124.5, 126.3 (2C), 129.7, 131.2, 133.2, 154.1, 156.3, 157.6. HR-MS analyst [M + H]^+^ *m*/*z* (predicted) = 295.1329, *m*/*z* (measured) = 295.1328, difference = 0.22 ppm.

#### 2.1.34. (*E*)-2-(4-Methoxyphenyl)-5-(prop-1-en-1-yl)benzo[*b*]furan (**20c**)

Prepared according to the general procedure D. mp 145–147 °C. ^1^H-NMR (400 MHz, CDCl_3_) δ (ppm) = 1.91 (dd, *J* = 6.6, 1.5 Hz, 3H), 3.86 (s, 3H), 6.22 (dq, *J* = 15.6, 6.5 Hz, 1H), 6.50 (dd, *J* = 15.7, 1.3 Hz, 1H), 6.83 (d, *J* = 0.6 Hz, 1H), 6.96–6.99 (m, 2H), 7.25–7.28 (m, 1H), 7.40–7.47 (m, 2H), 7.77–7.79 (m, 2H). ^13^C-NMR (100 MHz, CDCl_3_) δ (ppm) = 18.5, 55.4, 99.7, 110.9, 114.3 (2C), 117.7, 122.0, 123.4, 124.3, 126.4 (2C), 129.8, 131.2, 133.2, 154.1, 156.4, 160.0. HR-MS analyst [M + H]^+^ *m*/*z* (predicted) = 265.1223, *m*/*z* (measured) = 265.1223, difference = −0.01 ppm.

#### 2.1.35. (*E*)-2-(3,5-Dimethoxyphenyl)-5-(prop-1-en-1-yl)benzo[*b*]furan (**20d**)

Prepared according to the general procedure D. ^1^H-NMR (200 MHz, CDCl_3_) δ (ppm) = 1.91 (d, *J* = 6.4 Hz, 3H), 3.88 (s, 6H), 6.22 (dq, *J* = 15.9, 6.5 Hz, 1H), 6.47–6.55 (m, 2H), 6.97–7.03 (m, 3H), 7.27–7.50 (m, 3H). ^13^C-NMR (50 MHz, CDCl_3_) δ (ppm) = 18.5, 55.5 (2C), 101.0, 101.9, 102.9 (2C), 111.0, 118.0, 122.6, 124.6, 129.4, 131.1, 132.2, 133.3, 154.1, 156.1, 161.1 (2C). HR-MS analyst [M + H]^+^ *m*/*z* (predicted) = 313.1434, *m*/*z* (measured) = 313.1430, difference = 1.32 ppm.

#### 2.1.36. (*E*)-2-Phenyl-5-(prop-1-en-1-yl)benzo[*b*]furan (**20e**)

Prepared according to the general procedure D. mp 126–128 °C. ^1^H-NMR (200 MHz, CDCl_3_) δ (ppm) = 1.92 (dd, *J* = 6.4, 1.8 Hz, 3H), 6.22 (dq, *J* = 15.9, 6.5 Hz, 1H), 6.51 (d, *J* = 15.9 Hz, 1H), 7.00 (s, 1H), 7.27–7.52 (m, 6H), 7.87 (m, 2H). ^13^C-NMR (50 MHz, CDCl_3_) δ (ppm) =18.5, 101.3, 111.0, 118.0, 122.5, 124.5, 124.9 (2C), 128.5, 128.8 (2C), 129.5, 130.5, 131.1, 133.3, 154.2, 156.3. HR-MS analyst [M + H]^+^ *m*/*z* (predicted) = 235.1117, *m*/*z* (measured) = 235.1123, difference = −2.28 ppm.

#### 2.1.37. (*E*)-2-(4-Fluorophenyl)-5-(prop-1-en-1-yl)benzo[*b*]furan (**20f**)

Prepared according to the general procedure D. mp 123–125 °C. ^1^H-NMR (400 MHz, CDCl_3_) δ (ppm) = 1.91 (dd, *J* = 6.6, 1.5 Hz, 3H), 6.22 (dq, *J* = 15.6, 6.5 Hz, 1H), 6.50 (dd, *J* = 15.7, 1.3 Hz, 1H), 6.89 (s, 1H), 7.11–7.16 (m, 2H), 7.29–7.31 (dd, *J* = 8.6, 1.7 Hz, 1H), 7.41–7.49 (m, 2H), 7.80–7.83 (m, 2H). ^13^C-NMR (100 MHz, CDCl_3_) δ (ppm) = 18.5, 101.1, 111.0, 115.9 (d, ^2^*J*_C-F_ = 21.8 Hz), 118.0, 122.6, 124.6, 126.7 (d, ^3^*J*_C-F_ = 8.3 Hz), 126.8 (d, ^4^*J*_C-F_ = 3.2 Hz), 129.5, 133.4, 136.5, 154.2, 155.4, 161.3 (d, ^1^*J*_C-F_ = 247.2 Hz, 1C). HR-MS analyst [M + H]^+^ *m*/*z* (predicted) = 253.1023, *m*/*z* (measured) = 253.1027, difference = −1.41 ppm.

#### 2.1.38. (*E*)-2-(4-(Difluoromethyl)phenyl)-5-(prop-1-en-1-yl)benzo[*b*]furan (**20g**)

Prepared according to the general procedure D. mp 125–128 °C. ^1^H-NMR (200 MHz, CDCl_3_) δ (ppm) = 1.91 (dd, *J* = 6.6, 1.5 Hz, 3H), 6.23 (dq, *J* = 15.6, 6.5 Hz, 1H), 6.50 (dd, *J* = 15.7, 1.3 Hz, 1H), 6.68 (t, *J*_H-F_ = 56.4 Hz, 1H), 7.05 (s, 1H), 7.32–7.34 (m, 2H), 7.51–7.59 (m, 3H), 7.92 (d, *J* = 8.4 Hz, 2H). ^13^C-NMR (50 MHz, CDCl_3_) δ (ppm) = 18.5, 102.7, 111.2, 114.5 (t, ^1^*J*_C-F_ = 237.3 Hz), 118.2, 123.1, 124.8, 125.0 (2C), 126.1 (t, ^3^*J*_C-F_ = 6.2 Hz), 129.2, 131.0, 132.7, 133.5, 134.1 (t, ^2^*J*_C-F_ = 22.4 Hz), 154.4, 155.1. HR-MS analyst [M + H]^+^ *m*/*z* (predicted) = 285.1091, *m*/*z* (measured) = 285.1084, difference = 2.13 ppm.

#### 2.1.39. (*E*)-2-(2-Chlorophenyl)-5-(prop-1-en-1-yl)benzo[*b*]furan (**20h**)

Prepared according to the general procedure D. mp 66–68 °C. ^1^H-NMR (200 MHz, CDCl_3_) δ (ppm) = 1.94 (d, *J* = 6.5 Hz, 3H), 6.33 (dq, *J* = 15.6, 6.5 Hz, 1H), 6.55 (dd, *J* = 15.7, 1.3 Hz, 1H), 6.67–6.77 (m, 2H), 7.42–7.54 (m, 5H), 7.77–7.83 (m, 1H). ^13^C-NMR (50 MHz, CDCl_3_) δ (ppm) = 18.7, 105.6, 112.1, 120.4, 124.4, 125.1, 127.0, 127.4, 128.3, 128.5, 128.9, 129.0, 129.1, 130.0, 130.6, 153.0, 153.3. HR-MS analyst [M + H]^+^ *m*/*z* (predicted) = 269.0728, *m*/*z* (measured) = 269.0730, difference = −0.88 ppm.

#### 2.1.40. (*E*)-2-(4-(Methoxymethoxy)phenyl)-5-(prop-1-en-1-yl)benzo[*b*]thiophene (**25a**)

Prepared according to the general procedure D. mp 173–174 °C. ^1^H-NMR (400 MHz, CDCl_3_) δ (ppm) = 1.91 (dd, *J* = 6.6, 1.5 Hz, 3H), 3.50 (s, 3H), 5.21 (s, 2H), 6.28 (dq, *J* = 15.6, 6.5 Hz, 1H), 6.50 (dd, *J* = 15.8, 1.5 Hz, 1H), 7.08–7.10 (m, 2H), 7.32 (dd, *J* = 8.4, 2.6 Hz, 1H), 7.39 (s, 1H), 7.61–7.64 (m, 3H), 7.70 (d, *J* = 8.4 Hz, 1H). ^13^C-NMR (100 MHz, CDCl_3_) δ (ppm) = 18.5, 56.1, 94.4, 116.6 (2C), 118.5, 120.6, 122.1 (2C), 125.3, 127.7 (2C), 128.2, 131.1, 134.7, 137.7, 141.3, 144.4, 157.4. HR-MS analyst [M + H]^+^ *m*/*z* (predicted) = 310.1022, *m*/*z* (measured) = 310.1025, difference = −1.01 ppm.

#### 2.1.41. (*E*)-2-(4-Methoxyphenyl)-5-(prop-1-en-1-yl)benzo[*b*]thiophene (**25c**)

Prepared according to the general procedure D. mp 154–156 °C. ^1^H-NMR (200 MHz, CDCl_3_) δ (ppm) = 1.93 (d, *J* = 6.5 Hz, 3H), 3.86 (s, 3H), 6.29 (dq, *J* = 15.6, 6.5 Hz, 1H), 6.51 (d, *J* = 15.6 Hz, 1H), 6.96 (d, *J* = 8.6 Hz, 2H), 7.27–7.39 (m, 2H), 7.62–7.74 (m, 4H). ^13^C-NMR (50 MHz, CDCl_3_) δ (ppm) = 18.5, 55.4, 114.3 (2C), 118.2, 120.6, 122.0, 122.1, 125.2, 127.1, 127.7 (2C), 131.1, 134.6, 137.6, 141.3, 144.5, 159.8. HR-MS analyst [M + H]^+^ *m*/*z* (predicted) = 281.0995, *m*/*z* (measured) = 281.1004, difference = −3.48 ppm.

#### 2.1.42. General Procedure for De-Protection of MOM Group

Procedure D: MOM protected substrate (0.1 mmol, 1.0 equiv.) was dissolved in 0.5 mL MeOH. A 1N solution of HCl (50 µL, 0.05 mol, 0.5 equiv.) was added subsequently. The mixture was stirred at 65 °C for 30 min. The reaction mixture was cooled to room temperature and then diluted with 15 mL Et_2_O. The organic phase was washed with a saturated NH_4_Cl solution for 3 times, once with brine and dried over Na_2_SO_4_. The solvent was removed under reduced pressure. Purification was performed on silica gel eluting with LP/EtOAc 3:1 to obtain the desired product.

#### 2.1.43. 4-(5-Allylbenzo[*b*]furan-2-yl)phenol (**16b**)

Prepared according to the general procedure E. mp 165–168 °C. ^1^H-NMR (400 MHz, acetone-d6) δ (ppm) = 3.33 (d, *J* = 6.7 Hz, 2H, H-1″), 4.88–4.99 (m, 2H, H-3″), 5.88 (ddt, *J* = 16.8, 10.4, 6.7 Hz, 1H, H-2″), 6.82–6.84 (m, 2H), 6.89 (s, 1H), 6.96 (dd, *J* = 8.5, 1.2 Hz, 1H), 7.26–7.31 (m, 2H), 7.63–7.65 (m, 2H), 8.60 (s, 1H, ArOH). ^13^C-NMR (100 MHz, acetone-d6) δ (ppm) = 39.8 (C-1″), 99.1, 110.4, 114.7, 115.8 (2C), 120.2, 122.2, 124.5, 126.4 (2C), 129.9, 134.8, 138.3, 153.4, 156.6, 158.2. HR-MS analyst [M + H]^+^ *m*/*z* (predicted) = 251.1067, *m*/*z* (measured) = 251.1062, difference = 1.82 ppm.

#### 2.1.44. 4-(5-(3-Hydroxypropyl)benzo[*b*]furan-2-yl)phenol (**19b**)

Prepared according to the general procedure E. mp 194–196 °C. ^1^H-NMR (200 MHz, acetone-d6) δ (ppm) = 1.89 (ddt, *J* = 12.9, 8.9, 6.4 Hz, 2H), 2.80 (t, *J* = 7.2 Hz, 2H), 2.95 (s, 1H), 3.62 (t, *J* = 6.4 Hz, 2H), 6.95–6.97 (m, 3H), 7.10–7.13 (m, 1H), 7.39–7.40 (m, 2H), 7.75–7.77 (m, 2H), 8.66 (s, 1H). ^13^C-NMR (50 MHz, acetone-d6) δ (ppm) = 31.9, 35.1, 61.0, 99.1, 110.3, 115.7 (2C), 119.9, 122.2, 124.4, 126.3 (2C), 129.7, 137.0, 153.2, 156.5, 158.0. HR-MS analyst [M + H]^+^ *m*/*z* (predicted) = 269.1172, *m*/*z* (measured) = 269.1183, difference = −3.87 ppm.

#### 2.1.45. (*E*)-4-(5-(Prop-1-en-1-yl)benzo[*b*]furan-2-yl)phenol (**6**)

Prepared according to the general procedure E. mp 198–199 °C. ^1^H-NMR (200 MHz, acetone-d6) δ (ppm) = 1.87 (dd, *J* = 6.6, 1.2 Hz, 3H), 6.26 (dq, *J* = 15.6, 6.6 Hz, 1H), 6.51 (d, *J* = 15.8 Hz, 1H), 6.98–7.02 (m, 3H), 7.32 (dd, *J* = 8.6, 1.1 Hz, 1H), 7.44 (d, *J* = 8.5 Hz, 1H), 7.54 (s, 1H), 7.78–7.80 (m, 2H), 8.76 (s, 1H). ^13^C-NMR (50 MHz, acetone-d6) δ (ppm) = 17.7, 99.3, 110.6, 115.8 (2C), 117.7, 121.9, 122.1, 123.9, 126.5 (2C), 130.0, 131.2, 133.3, 153.9, 156.8, 158.2. HR-MS analyst [M + H]^+^ *m*/*z* (predicted) = 251.1067, *m*/*z* (measured) = 251.1057, difference = 3.71 ppm.

#### 2.1.46. 4-(5-(3-Hydroxypropyl)benzo[*b*]thiophen-2-yl)phenol (**24b**)

Prepared according to the general procedure E. mp 217–219 °C. ^1^H-NMR (400 MHz, acetone-d6) δ (ppm) = 1.74 (tt, *J* = 7.5, 6.2 Hz, 2H), 2.67 (t, *J* = 7.6 Hz, 2H), 2.73 (s, 1H), 3.46 (t, *J* = 5.8 Hz, 2H), 6.79–6.81 (m, 2H), 7.06 (dd, *J* = 8.2, 1.6 Hz, 1H), 7.38 (s, 1H), 7.47–7.65 (m, 4H), 8.54 (s, 1H). ^13^C-NMR (100 MHz, acetone-d6) δ (ppm) = 31.9, 34.9, 60.8, 115.9, 117.8, 121.8, 122.7, 125.1, 125.9, 127.6 (2C), 136.3, 139.0, 141.5, 144.4, 157.9. HR-MS analyst [M + H]^+^ *m*/*z* (predicted) = 285.0944, *m*/*z* (measured) = 285.0934, difference = 3.56 ppm.

#### 2.1.47. (*E*)-4-(5-(Prop-1-en-1-yl)benzo[*b*]thiophen-2-yl)phenol (**25b**)

Prepared according to the general procedure E. mp 212–213 °C. ^1^H-NMR (200 MHz, CDCl_3_) δ (ppm) = 1.74 (dd, *J* = 6.6, 1.6 Hz, 3H), 6.21 (dq, *J* = 15.7, 6.6 Hz, 1H), 6.38 (dd, *J* = 15.8, 1.5 Hz, 1H), 6.79–6.81 (m, 2H), 7.23 (dd, *J* = 8.4, 1.7 Hz, 1H), 7.37 (s, 1H), 7.47–7.49 (m, 2H), 7.56 (d, *J* = 1.2 Hz, 1H), 7.63 (d, *J* = 8.4 Hz, 1H), 8.54 (s, 1H). ^13^C-NMR (50 MHz, CDCl_3_) δ (ppm) = 17.8, 115.9 (2C), 117.9, 120.6, 122.0, 124.8, 125.7, 127.6 (2C), 127.6, 131.1, 134.8, 137.3, 141.6, 144.8, 158.0. HR-MS analyst [M + H]^+^ *m*/*z* (predicted) = 267.0838, *m*/*z* (measured) = 267.0830, difference = 3.22 ppm.

### 2.2. NF-κB Transactivation Activity

Measurement of the NF-κB transactivation activity was essentially performed as described previously [9]. Briefly, HEK-293 cells stably transfected with a NF-κB luciferase reporter (HEK293/NF-κB-luc cells, Panomics, RC0014) were loaded with CTG CMFDA (5-chloromethylfluorescein diacetate, Invitrogen) to stain living cells. 4 × 10^4^ cells were seeded in 96-well plates and incubated at 37 °C and 5% CO_2_ overnight. On the next day, the medium was exchanged with a serum-free DMEM and cells were treated with the respective test compounds dissolved in dimethyl sulfoxide (DMSO). To avoid nonspecific effects of the solvent, the final concentration of DMSO was always adjusted to 0.1%. One hour after the treatment, cells were stimulated with 2 ng/mL human recombinant TNF-α for 4 h. Then cells were lysed by a reporter lysis buffer (Promega, Madison, WI, USA). The luminescence of the firefly luciferase and the CTG fluorescence were quantified on a GeniosPro plate reader (Tecan, Grödig, Austria). The luciferase signal derived from the NF-κB reporter was normalized by the CTG-derived fluorescence to account for differences in the cell number or transfection efficiency.

## 3. Results and Discussion

**Figure 2:**
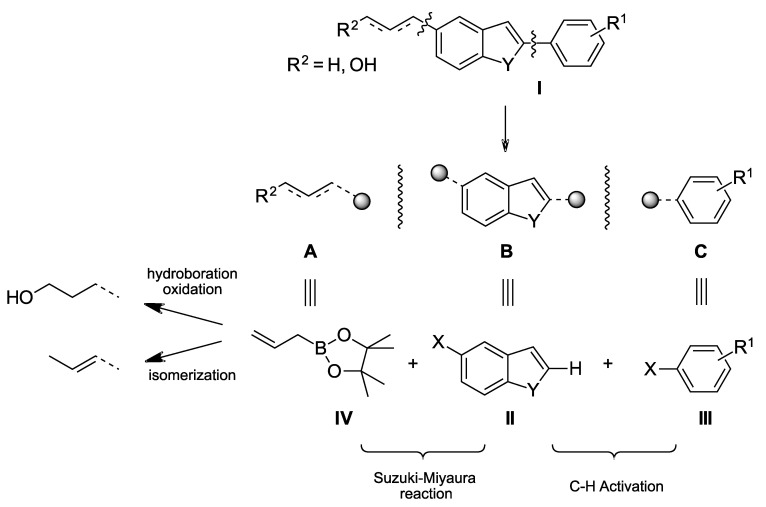
Retrosynthetic analysis.

Based on the results presented in the introduction, we were interested to synthesize and evaluate benzofuran-based lignans of general structure **I** (Figure 2). The synthetic strategy should be modular, efficient, and applicable to the synthesis of a wide range of derivatives, ideally from common intermediates. Breaking the strategic bonds as indicated in Figure 2, three fragments **A**, **B**, and **C**, are obtained which suggest the application of a direct arylation/cross coupling strategy, which introduces the aryl moiety in position two of general structure **II** via direct arylation and the alkyl or alkenyl chain in position five of the same fragment via a cross-coupling methodology, e.g., with an allyl boronic acid ester such as **IV**. For the direct arylation protocol we had previously developed an efficient protocol optimized for benzo-fused heterocyclic systems [10]. Hence, the main synthetic task was developing a suitable cross-coupling method introducing the desired residues in position five of building block **II** and subsequent elaboration of the olefin function towards the substituents identified for the naturally occurring compounds. Naturally, there are many ways to synthesize substituted benzofurans [11], and also the type of structure we are aiming for has been synthesized previously. For example, Duan et al. used a strategy in which the benzofuran core was constructed individually for each derivative synthesized [12]. This strategy is conveniently applicable for a small set of compounds, however, when a larger library of derivatives is targeted, it is lacking the modularity we were aiming for.

Consequently, fragment **B** was designed as a 5-halo-benzo[*b*]furan (general structure **II**, Figure 2). This building block offers two possibilities: Initial allylation at C5 and subsequent C2 arylation, or, alternatively, initial C2 arylation followed by C5-allylation. Since the direct arylation protocol uses aryl bromides as coupling partners, the allylation reaction should be carried out first to avoid homo-coupling between two 5-bromobenzofuran entities. The resulting 5-allyl benzofuran should then be arylated in position two, however it has to be considered that the terminal double bond could react in an undesired Mizoroki-Heck reaction. In case the order of events should be reversed, 5-chlorobenzofuran would need to be applied to avoid or at least suppress the aforementioned problem. To develop the most efficient protocol, it was decided to investigate both approaches. For introducing the side chain in position five, introduction of an allyl substituent is the ideal option, since the allyl substituent can be further transformed into 3-hydroxyl-propyl by a hydroboration-oxidation sequence [13,14,15] or to a 2-propenyl residue by an isomerization reaction [16,17,18,19,20,21,22,23].

**Scheme 1:**
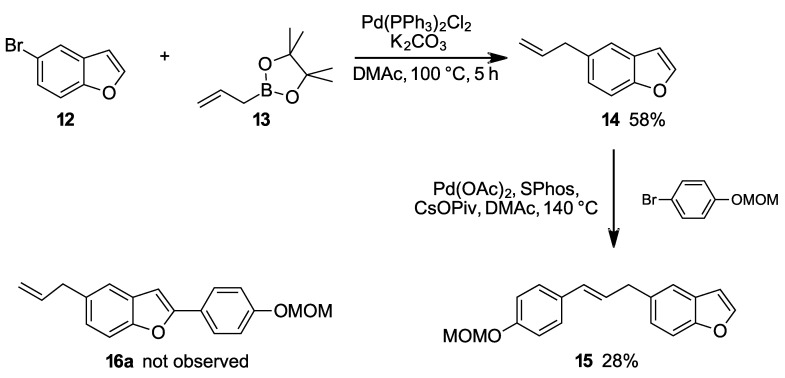
Allylation and attempted direct arylation of 5-bromobenzo[b]furan **12**.

Initially, 5-bromobenzo[*b*]furan **12** was tested as starting material (Scheme 1) [24,25,26,27]. The subsequent allylation reaction with **13** worked well using a Suzuki-Miyaura protocol with Pd(PPh_3_)_2_Cl_2_ as catalyst, K_2_CO_3_ as base and in DMAc solvent. Product **14** was obtained in 58% yield. Next, a direct arylation should introduce the aryl residue in position two, giving rise to **16**. Unfortunately, the direct arylation reaction did not take place and only product **15** derived from competing Mizoroki-Heck coupling was found, instead.

**Scheme 2:**
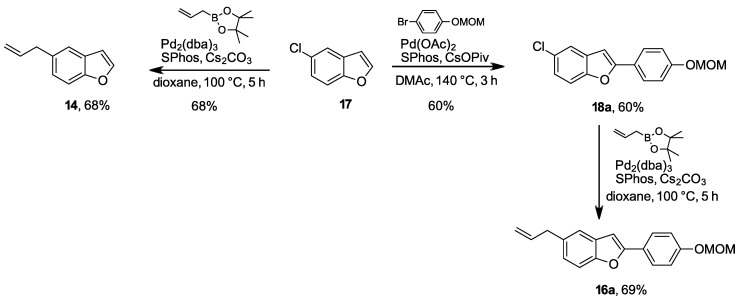
Allylation and attempted direct arylation of 5-chlorobenzo[b]furan **14**.

Hence, the alternative approach using 5-chlorobenzo[*b*]furan **17** as starting material was investigated (Scheme 2) [24,28]. In our previous publication [10], it was observed that aryl chlorides were unreactive as coupling partners under the reaction conditions and, hence, homo-coupling of the benzofuran starting material should not be an issue.

Before direct arylation was tested on this substrate, the allylation in position five had to be established. Using the same allylation conditions as for 5-bromobenzofuran **12** did not lead to any conversion towards **14** and, hence, the Suzuki-Miyaura reaction had to be optimized. Several conditions for Suzuki-Miyaura reactions on chloride substrates have been reported [29,30,31,32], mainly using elaborate catalyst/ligand systems. However, the method of Thimmaiah et al. used common catalysts and ligands and was therefore very appealing for our purposes of an efficient and simple protocol [30]. After a quick optimization, 5-allylbenzo[*b*]furan **14** could be synthesized using Pd_2_ (dba)_3_ as catalyst, SPhos as ligand, Cs_2_CO_3_ as base in dioxane, 100 °C and for 5 h giving an isolated yield of **14** of 68% (Scheme 2).

Subsequently, the direct arylation protocol was tested on 5-chlorobenzofuran **17** using our previously developed protocol [10]. Gratifyingly, **17** reacted selectively with 1-bromo-4-(methoxymethoxy) benzene giving product **18a** as sole compound in 60% yield after only three hours reaction time. Compound **18a** was subsequently submitted to the optimized Suzuki-Miyaura conditions to obtain product **16a** in 69% yield (Scheme 2).

**Scheme 3:**
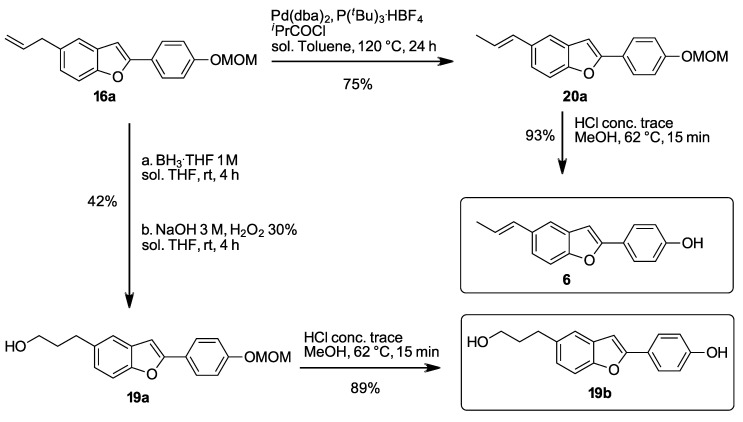
Overview of double bond isomerization, hydroboration–oxidation, and MOM-deprotection of **16a**.

Product **16a** represents a key compound for the synthetic route. From **16a**, a hydroboration-oxidation sequence [13,14,15] using BH_3_^.^THF 1M for the first step and NaOH, H_2_O_2_ in THF for the second step was utilized in situ to obtain alcohol **19a** with 42% isolated yield. The MOM protecting group was cleaved in methanol with traces of concentrated HCl subsequently to obtain the final product **19b** (Scheme 3).

For the isomerization of **16a** several different methods are reported using transition metal catalyst [16,17,18,19,20,21,22,23]. Only Gauthier et al. [23] used palladium catalyst with bulky ligand [23] to migrate the double bond into conjugation with the aryl ring with very good stereoselectivity. Using Pd(dba)_2_ as catalyst and P(*^t^*Bu)_3_^.^HBF_4_ as ligand product **20a** was obtained in *E* configuration with 75% yield. De-protection by concentrated HCl in methanol removed the MOM protective group to get final product **6** (Scheme 3).

**Scheme 4:**
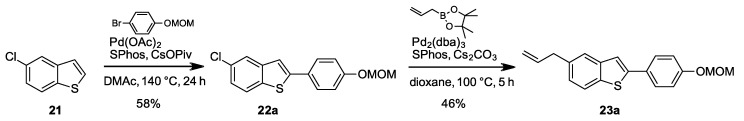
Direct arylation and allylation of 5-chlorobenzo[b]thiophene **21**.

For structure activity relationship studies, the developed synthetic sequence should also be applied for the synthesis of bioisosteric benzo[*b*]thiophene derivatives. Required 5-chlorobenzo[*b*]thiophene **21** was synthesized according to a literature protocol [33]. The direct arylation reaction on **21** was also selective giving **22a** in 58% yield. Allylation via Suzuki-Miyaura coupling on **22a** worked under the identical conditions as developed for the benzofuran series, albeit in somewhat lower yield giving **23a** in 46% (Scheme 4).

**Scheme 5:**
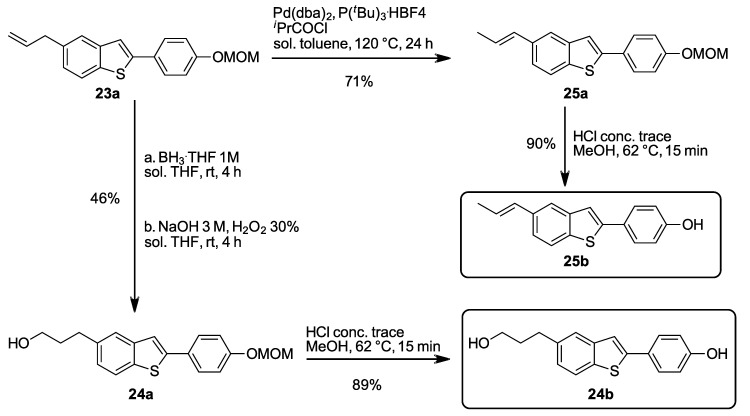
Overview of double bond isomerization, hydroboration–oxidation, and MOM-deprotection of **23a**.

The reactions for side chain modification were carried out again under identical conditions as in the benzofuran series. The hydroboration-oxidation sequence of **23a** gave 46% isolated yield of **24a** while isomerization to **25a** gave 71% yield. De-protection reaction on **24a** and **25a** proceeded in very good yield of 89% toward **24b** and 90% towards **25b**, respectively (Scheme 5).

With a practical synthesis route for benzo[*b*]furan and benzo[*b*]thiophene compounds at hand, a group of lignan-like compounds based on those heterocyclic rings was prepared to evaluate their biological properties as anti-inflammatory agents.

Initially, a series of direct arylation reactions was conducted (Table 1) with aryl moieties carrying electron donating and electron withdrawing substituents. The nature of the substituent had only a minor influence on the yield of this transformation. In the benzofuran series, aryl bromides carrying electron donating substituents such as OMOM or methoxy gave 10–15% higher yields in the arylation step (see Table 1 examples **18a**, **18c** and **18d**) as compared to substituents with no or an electron withdrawing effect (see Table 1 examples **18e**–**h**). In the benzothiophene series only two examples were synthesized and hence a general trend cannot be deduced.

The subsequent allylation reactions worked well on all benzofuran substrates giving yields in the range of 66–74%. Only the 2-Cl product **16h** was obtained in somewhat lower yield of 51%. Important to note: compound **16b** was obtained via MOM-deprotection of **16a** in 91% yield rather than via allylation of the corresponding 4-OH-aryl precursor **18b**. The direct arylation procedure turned out not to tolerate a free OH group, hence, requiring this alternate approach to **18b**.

The hydroboration-oxidation sequence towards the terminal alcohol products **19a**–**h** (benzofuran series) and **24a**–**c** (benzothiophene series) proceeded with similar efficiency (40–53% yield) independent of the substituents present on the aryl ring (Table 2, left). Again, it should be noted that the 4-OH products **19b** and **24b** were obtained in excellent yield via MOM-deprotection of **19a** and **24a** respectively. The same is true for the double bond isomerization (Table 2, right). Benzofuran products **20a**–**h** and benzothiophene compounds **25a**–**c** were obtained in yields between 57–77% yield. Also, in this case the 4-OH products **6** and **25b** were obtained in excellent yield via MOM-deprotection of **20a** and **25a**, respectively.

Since the pharmacological characterization of the lignan derivatives isolated from *Krameria lappaceae* revealed as most relevant in vitro anti-inflammatory activity inhibition of the NF-κB signaling pathway, we decided to use again a luciferase reporter model to quantify the transactivation activity of NF-κB [8]. For this, we used HEK293 cells stably transfected with a NF-κB-driven luciferase reporter gene that were loaded with fluorescent Cell Tracker Green to allow luciferase-derived signal normalization to the amount of viable cells. Cells were then treated with test compounds at the indicated concentration or vehicle for 30 min and then stimulated with TNF-α (2 ng/mL) for four hours. Luminescence and fluorescence was quantified in cell lysates by a Genios Pro plate reader (Tecan) [34].

## 4. Discussion

The biological data are compiled in Table 3. With the current synthetic route towards target compounds, structural diversity at position five of the benzo-heteroaromatic core could be further extended regarding the location of the olefinic system. Initially, 2-phenyl benzofurans were tested carrying an allyl group (entry 1, **16e**), a 1-propenyl group (entry 2, **20e**), or a 1-hydroxy-propan-3-yl group (entry 3, **19e**) in position five of the benzofuran scaffold. NF-κB inhibition was measured initially at concentrations of 10 μM and/or at 20 μM. IC_50_ values were determined for compounds with a significant and concentration-dependent inhibitory activity at these concentrations. Within this initial series, it was found that the propanol substituted derivative **19e** (entry 3) showed the highest inhibition (0.1-fold activation at 10 μM and 0.03-fold activation at 20 μM) and already a low IC_50_ value of 1.42 μM. The 5-allyl substituted derivative **16e** as well as the 5-(1-propenyl) derivative **20e** showed significantly lower inhibition, whereas the latter one gave the lowest activity (0.36 at 10 μM entry 1 vs. 0.66 at 10 μM entry 2).

Naturally occurring benzofuran lignans contain at least one oxygen functionality (OH or OMe) in the aryl ring in position two, compounds **16b**, **19b**, and **6** with a 4-hydroxyphenyl group in that position and the three different side chains in position five were tested (entries 4–6). Obviously, the hydroxyl group is very important for NF-κB inhibition since all three derivatives show significant inhibition at both, 10 and 20 μM concentration. Due to the small differences in the inhibitory effect, a trend between the three derivatives cannot be deduced, however, again the propanol substituted derivative **19b** (entry 6) showed the lowest IC_50_ value with 1.24 μM, which is also the lowest value of all tested compounds.

When a methoxy group is placed in position four of the phenyl ring instead of a hydroxy group, the trend that the highest inhibition is found with 5-propanol substituted benzofurans (entry 9, compound **19c**) followed by 5-allyl (entry 7, compound **16c**) and 5-propenyl (entry 8, compound **20c**) is clearly reestablished. Compound **19c** (entry 9) shows a similar inhibitory effect as the other two propanol substituted derivatives (entries 3 and 6 compounds **19e** and **19b**) but with a significantly higher IC_50_ value of 3.82 μM (vs. 1.42 entry 3, and 1.24 entry 6). Naturally, the methoxy group in **19c** is significantly larger than a proton (as in **19e**) or a hydroxy group (as in **19b**) and it was speculated that this size difference might have an influence on the IC_50_ values. Hence, in a next set of compounds, the methoxy group was substituted for a MOM group, which further increases the steric bulk.

In this set of compounds (entries 10–12, compounds **16a**, **20a**, and **19a** respectively), significant inhibitory effects were found with the 5-allyl- and 5-propanol substituted derivatives **16a** and **19a**, but only at the higher concentration of 20 µM. The 0.03-fold NF-κB activation of **19a** matches however the best values obtained so far. However, as hypothesized, the IC_50_ value is significantly higher with 9.22 μM (entry 12). Interestingly, **16a** gave a very low IC_50_ value of 1.31 μM. This supports the argument that steric bulk in the phenyl ring influences the IC_50_ values.

Since naturally occurring benzofuran lignans often carry two oxygen functionalities (OH, OMe), it was tried to access such compounds synthetically. Unfortunately, our synthetic method allowed us only access to 3,5-dimethoxy substituted derivatives **16d**, **20d**, and **19d** (entries 13–15). Again, the 5-propanol substituted derivative **19d** (entry 15) showed highest NF-κB inhibition (0.20 at 10 μM) and the overall highest NF-κB inhibition of 0.003 at 20 μM. The IC_50_ value for this compound was surprisingly low (1.92 μM, entry 15), which is contradicting the steric argument previously considered. However, this compound is the only one carrying a 3,5-disubstituted phenyl ring and additional derivatives incorporation this substitution pattern would be required to further establish structure-activity relationship. The two oxygen moieties might lead to favorable interactions, which predominate over steric effects.

At this point it is safe to say that a propanol substituent in position five usually gives highest NF-κB inhibition, which is sometimes matched by 5-allyl substituted derivatives. The propenyl derivatives are largely inactive and were excluded in further biological evaluation.

So far only electron donating substituents on the phenyl ring were considered, in line with the substitution pattern of the natural products. Since fluorine substituents often beneficially influence factors such as lipophilicity and hence also biological activity, two fluorine containing derivatives were tested as well (**19g** entry 16 and **19f** entry 17), both carrying the 5-propanol residue. NF-κB inhibition was mediocre in both compounds (entries 16 and 17) at 10 μM concentration, however at 20 μm, especially **19f** showed significant inhibition. The IC_50_ values again showed the trend that the larger 4-CHF_2_ substituent gave a significantly higher IC_50_ value (**19g**, entry 16, 8.52 μM) as compared to the 4-F substituted derivative (**19f**, entry 17, 2.20 μM).

In one example (**19h**), ortho substitution in the phenyl ring was tested as well, but no significant NF-κB inhibition was obtained (entry 18).

In a next series of compounds, we tested whether the benzofuran core could be substituted by other benzothiophene. Hence, several benzothiophene derivatives were synthesized and the 4-hydroxyphenyl- (**24b**, entry 19), 4-methoxyphenyl- (**24c**, entry 20) and 4-MOM-phenyl- (**24a**, entry 21) derivatives were tested. In all three examples position five was substituted by the propanol side-chain. The MOM substituted derivative did not show NF-κB inhibition (entry 21), which is surprising since the corresponding benzofuran derivative (entry 9) was amongst the most active ones. The 4-OH (entry 19) and 4-MeO (entry 20) derivative showed NF-κB inhibition, especially at the higher concentration of 20 μM, however the corresponding IC_50_ values were significantly higher as compared to their benzofuran counterparts (see entry 6 vs entry 19 and entry 9 vs. entry 20).

A comparison of the pharmacological data of our synthesized compounds and the benzofuran lignans isolated from *krameria lappacea* roots (Figure 1, compounds **1**, **2**, and **5**–**9**) shows that the IC_50_ values of several compounds are in the same range (or even lower) as the most active natural product **6**. For compound **6** an IC_50_ of 1.4 μM was reported in literature for the natural product isolate and we measured a similar value of 2.86 μM in our assay with a synthetic sample of **6**. Compound **19b**, which differs from **6** only in the sidechain (**19b**: 3-hydroxypropyl, **6**: prop-1-en-1-yl) gave an IC_50_ value of 1.24 μM. The difference between synthetic and natural **6** should not be overinterpreted, since the values stem from different measurement series carried out by different researchers.

## 5. Conclusions

It was found that the propanol side chain in position five is required for good inhibitory activity, independent of the underlying scaffold (benzofuran or benzothiophene). Some 5-allyl compounds do show activity as well, however those are the exception, and 5-propenyl derivatives are basically inactive. The IC_50_ values seem to correlate with steric bulk in the aryl-moiety in position two, a finding which we want to confirm further in subsequent studies. The larger the substituents get in this ring, the higher the IC_50_ values become. It can be speculated that there is a certain size restriction in the active site in this position. One examples does not follow this trend, however, in comparison to other compounds, the aryl ring is disubstituted in this example: the 3,5-dimethoxy compounds shows a low IC_50_ value, but other interactions induced by the two methoxy groups might be responsible for this. Here, more examples with different substitution patterns are required to complete the picture. With the established synthetic route towards this compound class, further elaboration of this scaffold has been enabled and additional studies to establish refined structure activity relationship will be conducted in our laboratories. The focus will lie on benzofurans carrying multiple oxygen-functionalities in the aromatic ring in position two. Additionally, further substitution in the benzofuran system besides a side chain in position five is not explored yet and will be investigated.

## Figures and Tables

**Table 1:** Direct arylation and subsequent allylation.

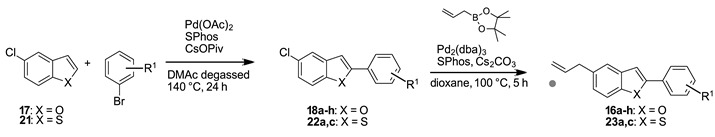						
Entry	X	R^1^	Product	Yield (%)	Product	Yield (%)
1	O	4-OMOM	**18a**	60%	**16a**	69%
2	O	4-OH	**18b**	n.s.	**16b**	91% ^1^
3	O	4-OMe	**18c**	58%	**16c**	70%
4	O	3,5-dimethoxy	**18d**	51%	**16d**	66%
5	O	H	**18e**	44%	**16e**	74%
6	O	4-F	**18f**	41%	**16f**	72%
7	O	4-CHF_2_	**18g**	45%	**16g**	69%
8	O	2-Cl	**18h**	44%	**16h**	51%
9	S	4-OMOM	**22a**	58%	**23a**	46%
10	S	4-OMe	**22c**	31%	**23c**	58%

n.s. not synthesized; ^1^ via MOM-deprotection from **16a**, the yield refers only to the deprotection step.

**Table 2:** Hydroboration-oxidation and isomerization.

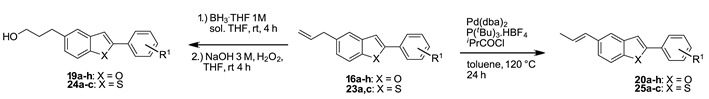						
Entry	X	R^1^	Product	Yield (%)	Product	Yield (%)
1	O	4-OMOM	**19a**	42%	**20a**	75%
2	O	4-OH	**19b**	89%^1^	**6**	93%^3^
3	O	4-OMe	**19c**	46%	**20c**	62%
4	O	3,5-dimethoxy	**19d**	40%	**20d**	77%
5	O	H	**19e**	52%	**20e**	75%
6	O	4-F	**19f**	53%	**20f**	77%
7	O	4-CHF_2_	**19g**	49%	**20g**	74%
8	O	2-Cl	**19h**	50%	**20h**	65%
9	S	4-OMOM	**24a**	46%	**25a**	71%
10	S	4-OH	**24b**	89%^2^	**25b**	90%^4^
11	S	4-OMe	**24c**	45%	**25c**	57%

^1^ the yield refers to the MOM-deprotection step of **19a**; ^2^ the yield refers to the MOM-deprotection step of **24a**; ^3^ the yield refers to the MOM-deprotection step of **20a**; ^4^ the yield refers to the MOM-deprotection step of **25a**.

**Table 3:** Pharmacological data.

Entry	Compound	Structure	Fold Activation NF-κB (10 µM)	Fold Activation NF-κB (20 µM)	IC_50_ (NF-κB, µM)
1	**16e**	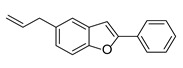	0.36	0.27	n.d.
2	**20e**	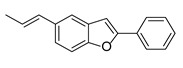	0.66	0.68	n.d.
3	**19e**	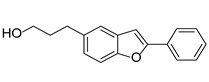	0.10	0.03	1.46
4	**16b**	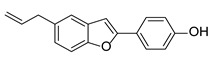	0.06	0.02	2.12
5	**6**	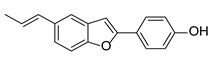	0.15	0.04	2.86
6	**19b**	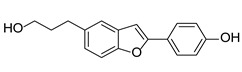	0.12	0.007	1.24
7	**16c**	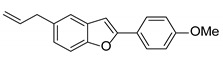	0.60	0.28	3.60
8	**20c**	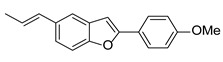	n.d.	0.78	n.d.
9	**19c**	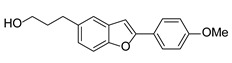	0.12	0.04	3.82
10	**16a**	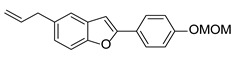	0.31	0.147	1.31
11	**20a**	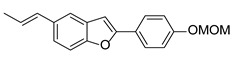	0.77	0.83	n.d.
12	**19a**	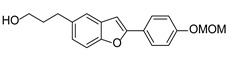	0.57	0.03	9.22
13	**16d**	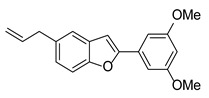	0.54	n.d.	n.d.
14	**20d**	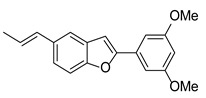	0.94	n.d.	n.d.
15	**19d**	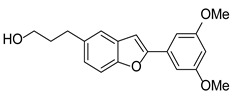	0.20	0.003	1.92
16	**19g**	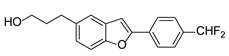	0.52	0.08	8.52
17	**19f**	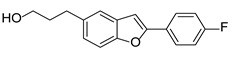	0.32	0.02	2.20
18	**19h**	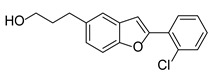	1.08	0.61	n.d.
19	**24b**	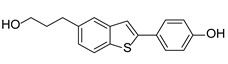	0.21	0.05	4.74
20	**24c**	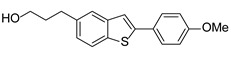	0.37	0.12	6.59
21	**24a**	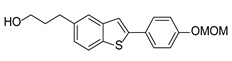	0.96	1.08	n.d.

Compounds were tested at 10 µM and/or at 20 µM in a luciferase-based cell model (HEK293/NF-κB-luc cells) for NF-κB Inhibition. Values display residual fold activation after treatment with the indicated compounds (n = 3; with TNF-α-induced activation set to 1); for selected compounds an IC_50_ value was determined; n.d.: not determined.
